# Treatment of Naoxueshu Promotes Improvement of Hematoma Absorption and Neurological Function in Acute Intracerebral Hemorrhage Patients

**DOI:** 10.3389/fphys.2018.00933

**Published:** 2018-07-20

**Authors:** Juexian Song, Yi Lyu, Pingping Wang, Yuting Nie, Huiqiang Lu, Li Gao, Xiaolin Tong

**Affiliations:** ^1^Department of Neurology, Xuanwu Hospital, Capital Medical University, Beijing, China; ^2^Baoshan Anli Hospital, Baoshan, China; ^3^The Key Laboratory of Development Biology, College of Life Science, Jinggangshan University, Jinggangshan, China; ^4^Guang'anmen Hospital, China Academy of Chinese Medical Sciences, Beijing, China

**Keywords:** Naoxueshu oral liquid, intracerebral hemorrhage, hematoma, neurological function, NIHSS

## Abstract

**Aims:** To evaluate the clinical efficacy of Naoxueshu oral liquid in the treatment of intracerebral hemorrhage (ICH) patients.

**Methods:** In our study, December 2008 to August 2010, 88 patients with intracerebral hemorrhage were enrolled and 87 patients with complete information of whom 44 patients received Naoxueshu oral liquid plus regular treatment (Naoxueshu group), 43 patients received regular treatment (control group) only. Naoxueshu oral liquid 10 ml was taken in the Naoxueshu group, with 3 times a day for 21 consecutive days. The regular treatment included (1) dehydration treatment by 20% mannitol; (2) therapy to deal with complications including; (3) supportive therapy. The general clinical information, neurological assessment information, laboratory information, and the hematoma volume information were collected and analyzed pre-and post-treatment.

**Results:** We did not find differences in the information between two groups before treatment (*p* > 0.05). 21-day after treatment, the white blood cell (WBC) count, hematoma volume, the National Institutes of Health Stroke Scale (NIHSS) score, modified Rankin Scale (mRS), Barthel index (BI), and traditional Chinese medicine (TCM) syndrome score in the Naoxueshu group and control group were significantly decreased than before (*p*_naoxueshu_ < 0.01, *p*_control_ < 0.05), and the changes of the WBC count, hematoma volume, NIHSS score, mRS score, and TCM syndrome score in Naoxueshu group were greater than that of control group (*P* < 0.001).

**Conclusion:** Naoxueshu oral liquid plus regular treatment could decrease the inflammatory response and hematoma, and improve outcomes of ICH patients than regular treatment only. This suggests that Maixueshu oral liquid is a potential treatment for ICH patients.

## Introduction

Intracerebral hemorrhage (ICH) is a common kind of stroke. It accounts for 10–15% of all stroke types worldwide. There is a significant difference among different races. In Asia, ICH accounts for 20–30% of all stroke patients and it accounts for 18.8–47.6% of hospitalized stroke patients (Arias-Rivas et al., 2012; Klionsky et al., 2016). The mortality rate of ICH is high and the outcome is worse. The mortality of ICH in 3 months is 20~30%, which caused a heavy burden to the society and the family (Feigin et al., 2014). Currently, the mechanism of ICH has not been fully elucidated, and the therapy of ICH mainly depends on the mannitol dehydration treatment but lacks of neuroprotective drugs. Exploring effective therapy for ICH is still urgent challenge for stroke researchers.

Nowadays, more and more traditional Chinese medicine (TCM) studies are focusing on the therapy of stroke (Wu et al., 2007), but only a few of them are carried out for searching therapies for ICH (Gao et al., 2012). Naoxueshu oral liquid mainly includes leech, astragalus root, Rhizome Chuanxiong calamus, and Achyranthes. It has the effect of replenishing Qi and activate blood and removing blood stasis and it is mainly used for hemorrhagic stroke in patients with Qi deficiency and blood stasis (Chen et al., 2014). Leech has a broken blood and blood stasis eliminating effect; modern researches show that leech has a cerebral protective effect of anticoagulation, inhibiting platelet aggregation, improving blood rheology, and relieving acute brain injury and brain edema, among other benefits (Li et al., 2007; Zhang et al., 2007; Dong et al., 2016). Astragalus is commonly used in TCM Yiqi, and modern researches show that astragalus has the ability of reducing brain damage after hemorrhage, inhibiting neuronal apoptosis, and promoting the recovery of neurological function. In addition, astragalus can also protect the blood-brain barrier permeability, perform antioxidation, and prevent cerebral ischemia (Liu et al., 2012; Wang et al., 2013; Cai et al., 2014). Previous studies found that Naoxueshu oral liquid could improve aphasia in mixed stroke patients (Yan et al., 2015) and it could also protect against the occurrence of secondary brain insults and hypertensive cerebral hemorrhage (Jiang et al., 2016). Whether it could be useful in the treatment of ICH is still unknown. Therefore, we designed this clinical trial to explore the efficiency of Naoxueshu oral liquid in the ICH therapy.

## Materials and methods

### Patients

This was a random, single-blind, multicenter, prospective study approved by the Ethics Committee of the Xuanwu Hospital of Capital Medical University, China. From December 2008 to August 2010, 88 patients with ICH were selected from 6 hospitals in Beijing, China. All patients signed informed consent before treatment. All the patients were evaluated by the same group of trained neurologists.

Patients in this study at the onset of their first acute ischemic stroke, were confirmed by magnetic resonance imaging (MRI) or computer tomography (CT), Inclusion criteria were: (1) patients were in accordance with the diagnostic criteria of cerebral infarction approved by the fourth national cerebrovascular academic conference (1995) and the criteria of diagnosis and curative effect evaluation of stroke (Trial) (State Administration of traditional Chinese medicine, 1996); (2) 18 to 75 years old; (3) intracerebral hemorrhage within 7 days (including 7 days); (4) no loss of consciousness; no incomplete hepatic or renal function or severe psychotic disease; (5) NIHSS score between 2 and 24; (6) obtain informed consent from the patients or their legal surrogates.

### Patients groups and therapeutic methods

All patients were randomly divided into two groups: Naoxueshu group (Naoxueshu oral liquid 10 ml, 3 times a day plus regular treatment) and control group (regular treatment). Naoxueshu oral liquid 10 ml was taken orally 3 times a day for at least 3 weeks. Regular treatment was defined as: (1) dehydration treatment by 20% mannitol; (2) therapy to deal with complications including; (3) supportive therapy.

### Information collection

Basic information: age, gender, body mass index (BMI), previous disease history (hypertension, diabetes, heart disease, and hyperlipidemia), and personal history (smoking and alcohol consumption). General clinical information: temperature, heart rate, systolic pressure, and diastolic pressure. Neurological assessment information: the National Institutes of Health Stroke Scale (NIHSS) score, Barthel index (BI), modified Rankin Scale (mRS), and TCM syndrome score. Laboratory information: red blood cell (RBC) count, white blood cell (WBC) count, prothrombin time (PT), activated partial thromboplastin time (APTT), fibrinogen (FIB), alanine aminotransferase (ALT), aspartate aminotransferase (AST), blood urea nitrogen (BUN), creatinine (Cr), platelet maximum aggregation rate (PAgT), C-reaction protein (CRP). Hematoma information: hematoma volume (ml) = π/6 × length (cm) × width (cm) × high (cm), was determined by CT scanning (Siemens 64-slice CT machine, section thickness, 5 mm; gap, 5 mm; pitch, 1; tube current, 304 mA; and voltage, 120 kV).

### Statistical analyses

Statistical analyses were performed using SPSS17.0 software (IBM SPSS, Armonk, NY, USA). Continuous variables were expressed as a mean ± standard deviation (SD). Categorical variables were analyzed by Pearson Chi-square test. Factors of pre-and post-treatment were analyzed by paired-samples *t*-test and the independent-samples *t*-test was used to compare factors in different groups. *P* < 0.05 was considered to be statistically significant.

## Results

**Figure 1:**
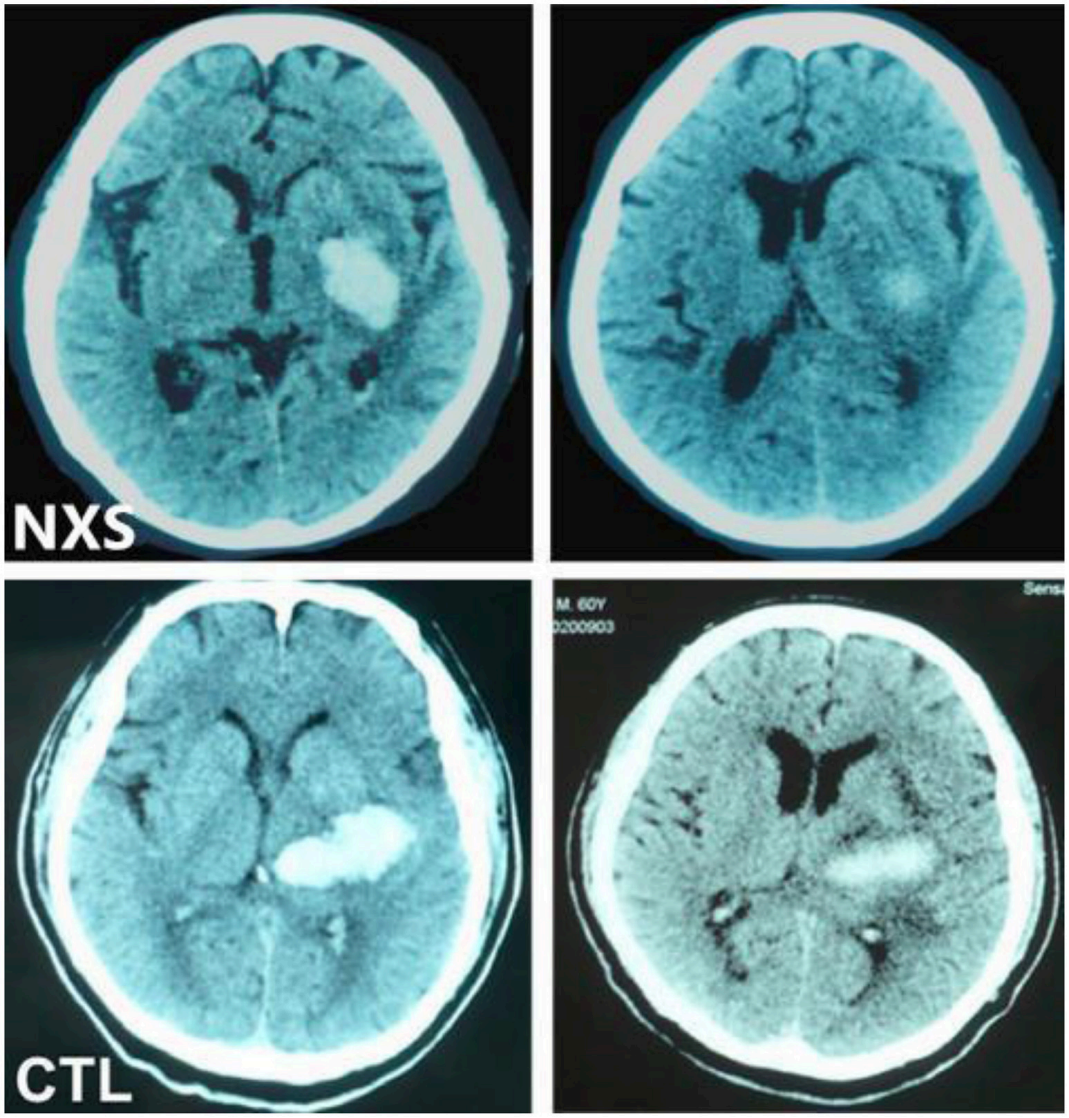
Comparison of changes in hematoma volume between two patients. **Left**: pre-treatment; **Right**: post-treatment; Representative CT images of hematoma absorption progress from hemorrhagic stroke patients with or without Naoxueshu treatment. Both patients, at age of 71 (Naoxueshu group, NXS) and 68(Control group, CTL), were admitted as acute hypertensive intracerebral hemorrhage, with hematoma volume of 25 ml (NXS group) and 26 ml (CTL group) and received regular therapy, with (NXS group) or without (CTL group) supplementary Naoxueshu treatment. Naoxueshu oral liquid promoted the hematoma absorption after a 21-day treatment.

Eighty-eight patients were enrolled and randomly divided either to the Naoxueshu group (*n* = 44) or control group (*n* = 44), one patient in the control group was lost to follow-up (Figure 1). The Naoxueshu group had 31 male and 13 female, the average age was 58.22 ± 12.33 years; and the control group had 23 male and 20 female, the average age was 62.93 ± 10.05 years.

### Comparison of basic information and stroke risk factors

There was no statistical difference in age and sex between the groups (*P* > 0.05). The history of smoking, drinking, hypertension, heart disease, hyperlipidemia, and diabetes were considered to be the main risk factors for stroke and no statistical difference of these factors was found between the two groups (*P* > 0.05) (Table 1).

**Table 1:** Comparison of basic information and stroke risk factors.

**Factors**	**Naoxueshu group (*n* = 44)**	**Control group (*n* = 43)**	***P-*value**
Age (years)	58.22 ± 12.33	62.93 ± 10.05	0.06
Sex	31(70.45%)	23(53.49)	0.18
BMI	24.86 ± 5.78	25.04 ± 5.92	0.54
Hypertension	27(61.36%)	26(60.47%)	0.68
Heart disease	9(20.45%)	10(23.26%)	0.75
Hyperlipidemia	6(13.64%)	7(16.28%)	0.73
Diabetes	31 70.45%)	11(25.58%)	0.68
Smoking	19(43.18%)	20(46.51%)	0.76
Alcohol consumption	12(73.78%)	10(23.26%)	0.67

### Comparison of general clinical information before treatment

No statistical difference was observed between the two groups in BMI, body temperature, heart rate, or blood pressure before treatment (*P* > 0.05). There was no statistical significance in the NIHSS score, mRS, BI score and TCM syndrome score before treatment (*P* > 0.05). There was also no statistical difference in laboratory data of hematoma volume before treatment (*P* > 0.05) (Table 2).

**Table 2:** Comparison of general clinical information before treatment.

**Factors**	**Naoxueshu group (*n* = 44)**	**Control group (*n* = 43)**	***P-*value**
Temperature (°C)	36.55 ± 0.34	36.57 ± 0.47	0.79
Heart rate (*n*/min)	76.16 ± 6.71	75.65 ± 6.95	0.73
Systolic pressure (mmHg)	159.18 ± 28.36	152.21 ± 22.58	0.21
Diastolic pressure (mmHg)	94.04 ± 18.86	87.57 ± 11.38	0.06
Hematoma volume (ml)	16.26 ± 10.05	17.12 ± 9.49	0.72
NIHSS score	6.89 ± 4.01	7.44 ± 5.47	0.59
mRS	3.93 ± 1.42	4.00 ± 1.43	0.83
BI	49.84 ± 26.08	45.58 ± 28.01	0.46
TCM syndrome score	10.11 ± 5.04	9.05 ± 4.29	0.29
RBC (× 10^∧^12)	4.75 ± 0.47	4.66 ± 0.46	0.37
WBC (× 10^∧^9)	7.87 ± 2.19	8.15 ± 2.19	0.56
PT(s)	20.96 ± 7.45	18.21 ± 9.06	0.21
TT(s)	27.63 ± 14.23	29.83 ± 14.88	0.11
APTT(s)	33.12 ± 6.98	30.45 ± 7.54	0.09
FIB (g/L)	3.38 ± 1.01	3.14 ± 0.99	0.27
ALT (U/L)	22.89 ± 8.74	19.35 ± 6.92	0.25
AST (U/L)	26.27 ± 6.41	23.98 ± 7.35	0.40
BUN (mmol/L)	4.97 ± 4.74	5.91 ± 6.14	0.42
Cr (umol/L)	68.13 ± 19.94	81.40 ± 13.39	0.07
PAgT(s)	56.03 ± 15.16	55.30 ± 11.94	0.8
CRP (mg/L)	2.68 ± 1.33	3.56 ± 1.25	0.07

*NIHSS, National Institutes of Health Stroke Scale; mRS, modified Rankin Scale; BI, Barthel index; TCM, traditional Chinese medicine; RBC, red blood cell; WBC, white blood cell; PT, prothrombin time; APTT, activated partial thromboplastin time; FIB, fibrinogen; ALT, alanine aminotransferase; AST, aspartate aminotransferase; BUN, blood urea nitrogen; Cr, creatinine; PAgT, platelet maximum aggregation rate; CRP, C-reaction protein*.

### Comparison of baseline data and the data of the 21st day after treatment

The hematoma volume, NIHSS score, mRS, BI, and TCM syndrome score were significantly reduced on the 21st day after treatment in two groups (*P* < 0.05); the level of WBC was reduced on the 21st day after treatment (*P*_Naoxueshu_ < 0.01, *P*_control_ < 0.01); but the level of Fib was elevated (*P*_Naoxueshu_ < 0.01, *P*_control_ < 0.01), so were the level of ALT (*P*_Naoxueshu_ < 0.01, *P*_control_ < 0.01, and AST (*P*_Naoxueshu_ < 0.01, *P*_control_ < 0.04) (Table 3).

**Table 3:** Comparison of baseline data and the data of the 21st day after treatment.

	**Naoxueshu group (*n* = 44)**			**Control group (*n* = 43)**		
**Factors**	**Baseline data**	**21st day data**	***P-*value**	**Baseline data**	**21st day data**	***P-*value**
Hematoma volume (ml)	16.26 ± 10.05	2.66 ± 3.40	<0.01	17.12 ± 9.49	5.16 ± 6.17	<0.01
NIHSS score	6.89 ± 4.01	3.64 ± 3.10	<0.01	7.44 ± 5.47	4.55 ± 4	<0.01
mRS	3.93 ± 1.42	3.03 ± 1.61	<0.01	4.00 ± 1.43	3.48 ± 1.43	0.04
BI	49.84 ± 26.08	66.20 ± 25.60	<0.01	45.58 ± 28.01	63.10 ± 29.61	<0.01
TCM Syndrome score	10.11 ± 5.04	3.42 ± 2.59	<0.01	9.05 ± 4.29	4.40 ± 2.31	<0.01
RBC (× 10^∧^12)	4.75 ± 0.47	4.89 ± 1.09	0.41	4.66 ± 0.46	4.57 ± 0.45	0.09
WBC (× 10^∧^9)	7.87 ± 2.19	6.91 ± 1.41	<0.01	8.15 ± 2.19	6.99 ± 1.79	<0.01
PT(s)	20.96 ± 7.45	18.73 ± 9.45	0.80	18.21 ± 9.06	15.34 ± 8.06	0.63
TT(s)	27.63 ± 14.23	18.15 ± 10.59	0.13	29.83 ± 14.88	19.11 ± 14.91	0.16
APTT(s)	33.12 ± 6.98	33.28 ± 7.25	0.16	30.45 ± 7.54	31.11 ± 5.85	0.28
FIB (g/L)	3.38 ± 1.01	4.09 ± 1.19	<0.01	3.14 ± 0.99	4.00 ± 1.32	<0.01
ALT (U/L)	22.89 ± 8.74	40.23 ± 7.85	<0.01	19.35 ± 6.92	27.43 ± 10.9	<0.01
AST (U/L)	26.27 ± 6.41	31.62 ± 11.87	0.01	23.98 ± 7.35	27.43 ± 10.79	0.04
BUN (mmol/L)	4.97 ± 4.74	6.59 ± 8.17	0.25	5.91 ± 6.14	5.46 ± 2.39	0.65
Cr (μmol/L)	68.13 ± 19.94	64.81 ± 13.90	0.12	81.40 ± 13.39	74.65 ± 18.81	0.15
PAgT(s)	56.03 ± 15.16	53.74 ± 15.43	0.46	55.30 ± 11.94	56.69 ± 14.45	0.56
CRP (mg/L)	2.68 ± 1.33	2.50 ± 1.20	0.56	3.56 ± 1.25	2.41 ± 1.44	0.61

*NIHSS, National Institutes of Health Stroke Scale; mRS, modified Rankin Scale; BI, Barthel index; TCM, traditional Chinese medicine; RBC, red blood cell; WBC, white blood cell; PT, prothrombin time; APTT, activated partial thromboplastin time; FIB, fibrinogen; ALT, alanine aminotransferase; AST, aspartate aminotransferase; BUN, blood urea nitrogen; Cr, creatinine; PAgT, platelet maximum aggregation rate; CRP, C-reaction protein*.

### Comparison of the data of the 21st day after treatment between two groups

Observations in our pilot clinical trial revealed that patients in Naoxueshu group demonstrated facilitated hematoma absorption and reduced brain edema, as shown in Figure 1. In addition, the NIHSS score, mRS, and TCM syndrome score of Naoxueshu group were significant lower than that of control group (*P* < 0.05). There was no statistical difference in BI between two groups (*P* = 0.60). No statistical significance was observed in other factors (Table 4).

**Table 4:** Comparison of the data of the 21st day after treatment between two groups.

**Factors**	**Naoxueshu group (*n* = 44)**	**Control group (*n* = 43)**	***P-*value**
Hematoma volume	2.66 ± 3.40	5.16 ± 6.17	0.04
NIHSS score	3.64 ± 3.10	4.55 ± 4	0.01
mRS	3.03 ± 1.61	3.38 ± 1.43	0.04
BI	66.20 ± 25.60	63.10 ± 29.61	0.60
TCM Syndrome score	3.42 ± 2.59	4.40 ± 2.31	0.02
RBC (× 10^∧^12)	4.89 ± 1.09	4.57 ± 0.45	0.08
WBC (× 10^∧^9)	6.91 ± 1.41	6.99 ± 1.79	0.82
PT(s)	18.73 ± 9.45	15.34 ± 8.06	0.35
TT(s)	18.15 ± 10.59	19.11 ± 14.91	0.06
APTT(s)	33.28 ± 7.25	31.11 ± 5.85	0.18
FIB (g/L)	4.09 ± 1.19	4.00 ± 1.32	0.72
ALT (U/L)	40.23 ± 7.85	27.43 ± 10.9	0.06
AST (U/L)	31.62 ± 11.87	27.43 ± 10.79	0.09
BUN (mmol/L)	6.59 ± 8.17	5.46 ± 2.39	0.39
Cr (umol/L)	64.81 ± 13.90	74.65 ± 18.81	0.08
PAgT(s)	53.74 ± 15.43	56.69 ± 14.45	0.36
CRP (mg/L)	2.50 ± 1.20	2.41 ± 1.44	0.56

*NIHSS, National Institutes of Health Stroke Scale; mRS, modified Rankin Scale; BI, Barthel index; TCM, traditional Chinese medicine; RBC, red blood cell; WBC, white blood cell; PT, prothrombin time; APTT, activated partial thromboplastin time; FIB, fibrinogen; ALT, alanine aminotransferase; AST, aspartate aminotransferase; BUN, blood urea nitrogen; Cr, creatinine; PAgT, platelet maximum aggregation rate; CRP, C-reaction protein*.

Furthermore, NIHSS score of 60th day in Naoxueshu Group is 2.40 ± 2.27 was significant lower than that in Control Group which is 3.69 ± 3.52. NIHSS score of 90th day in Naoxueshu Group is 1.78 ± 2.17 was also significant lower than that in Control Group which is 3.14 ± 3.3.

## Discussion

In this study, we investigated the effects of the Naoxueshu oral liquid in hypertensive patients with intracerebral hemorrhage. Patients who were given Xueshuantong oral liquid treatment showed significantly better improvement in behavioral function recovery and hematoma absorption. After a 21-day treatment, the WBC count, hematoma volume, NIHSS score, mRS, and TCM syndrome score of the Naoxueshu group was significant decreased when compared with the control group. Therefore, patients in Naoxueshu group had a better recovery of neurological functions than patients in control group.

Intracerebral hemorrhage (ICH) is a kind of common neurological disorder, has an estimated incidence of more than 37,000 cases per year (Thompson et al., 2007). Knowledge of effective treatment is still limited, especially for those with hypertension. Because the main limitation in ICH therapy is the management of increased blood pressure (Morgenstern et al., 2010). Therefore, exploring effective therapy for ICH is still urgently needed. Previously, the voluable efficiency of the TCM have been showed in animal studies with a rat model of cerebral hemorrhage (Zhang et al., 2007), and it has demonstrated that Naoxueshu oral liquid was effective in the treatment of ICH rats, which could significantly promote the absorption of hematoma and restore the nerve function of rats (Liu, 2015).

Naoxueshu oral liquid is a crude extraction from the TCM, which has the function of activating blood and removing stasis. The main components are leech, ligusticum chuanxiong, acorus calamus, achyranthes, peony skin, and rhubarb. Hirudo, the active ingredient in Hirudo, can prolong or inhibit the coagulation process, and it has the effect of promoting fibrinolysis and the thrombin induced blood coagulation is the main mechanism of vascular thrombosis. Ligusticum chuanxiong can pass the blood-brain barrier, relieve the vasospasm caused by adrenaline, expand blood vessels, improve microcirculation, increase blood flow velocity, reduce blood viscosity and improve blood microcirculation (Kang et al., 2015). The beneficial effect of Naoxueshu oral liquid in ICH patients could be explained by several mechanisms. Pharmacological researches show that Naoxueshu oral liquid has effect of anti-coagulation, thrombin inhibition, anti-platelet aggregation, promoting fibrinolysis, increasing cerebral blood flow, reducing cerebrovascular resistance, relieving smooth muscle spasm, and improving neurological symptoms of cerebral infarction.

Previous studies have reported that there are many inflammatory responses following cerebral hemorrhage, such as leukocyte infiltration, the activation of the complement system, microglial cell activation, and cytokines expression (Hallenbeck et al., 2005; Florczak-Rzepka et al., 2012). In this study, we investigated the changes of inflammatory factors in ICH patients after Naoxueshu oral liquid treatment. The results showed that changes in CRP level were not significant in both groups, while the level of WBC count, was significantly decreased in patients after treatment, especially in Naoxueshu group. These findings indicate that the Naoxueshu oral liquid treatment may be an effective neuroprotective therapy through the mechanisms of anti-inflammatory in patients with ICH. In addition, we also found the level of fib, ALT and AST were elevated after 21-days treatment in Naoxueshu group and control group, and no significant difference was found between two groups. This may because after cerebral hemorrhage, the release of thrombin combined with fibrin increase secondarily, which could accelerate the blockage of the ruptured blood vessels and block the rebleeding. The elevated thrombin and fibrin would cause the increase of ALT and AST levels (Tan et al., 2016).

The volume of hematoma is an important predictor of prognosis for ICH. Previous studies have shown that stabilizing hematoma growth and reducing the volume of hematoma could improve outcomes in ICH patients (Nyquist, 2010). The results in this study showed that the hematoma in the Naoxueshu group was significantly reduced after treatment and the changes between pre- and post-treatment for the hematoma was significantly greater than that of the control group. Furthermore, the NIHSS score, mRS, and TCM syndrome score, which can reflect the severity of stroke and the functional condition, were decreased greater in Naoxueshu group than that of control group. These results indicated that Naoxueshu oral liquid could help the absorption of hematoma and improve the outcome of ICH patients.

This study has several limitations: First of all, it was a small scale study, which we can't get enough data to go subgroup analysis. Then, there was no classification of ICH types according to the location of hematoma, which may have influence on the results. Moreover, we did not record the changes of blood pressure, which may reflect the association with the hematoma volume, and the difference in diastolic pressure between the two groups was close to significance (*P* = 0.06), we could not completely exclude whether the diastolic pressure can influence the results. In the end, patients in Naoxueshu group received Naoxueshu oral liquid while patients in control group received no additional treatment, we also could not exclude the placebo effect. Therefore, in the future, we need additional studies to clarify these problems.

In conclusion, the results in our study demonstrated that treatment of Naoxueshu oral liquid could reduce the hematoma and inflammatory response, and improve outcomes of ICH patients. This suggests that Naoxueshu oral liquid is a potential effective treatment for ICH patients.

## Author contributions

LG and XT designed the study. JS and YL wrote the manuscript. JS, PW, and YN managed the study. HL done the data analysis.

### Conflict of interest statement

The authors declare that the research was conducted in the absence of any commercial or financial relationships that could be construed as a potential conflict of interest.
